# A straightforward multiallelic significance test for the Hardy-Weinberg equilibrium law

**DOI:** 10.1590/S1415-47572009000300028

**Published:** 2009-09-01

**Authors:** Marcelo S. Lauretto, Fabio Nakano, Silvio R. Faria, Carlos A. B. Pereira, Julio M. Stern

**Affiliations:** 1 Escola de Artes, Ciências e Humanidades, Universidade de São Paulo, São PauloBrazil; 2 Instituto de Química, Universidade de São Paulo, São PauloBrazil; 3 Instituto de Matemática e Estatística Universidade de São Paulo, São PauloBrazil

**Keywords:** Hardy-Weinberg equilibrium, significance tests, FBST

## Abstract

Much forensic inference based upon DNA evidence is made assuming Hardy-Weinberg Equilibrium (HWE) for the genetic loci being used. Several statistical tests to detect and measure deviation from HWE have been devised, and their limitations become more obvious when testing for deviation within multiallelic DNA loci. The most popular methods-Chi-square and Likelihood-ratio tests-are based on asymptotic results and cannot guarantee a good performance in the presence of low frequency genotypes. Since the parameter space dimension increases at a quadratic rate on the number of alleles, some authors suggest applying sequential methods, where the multiallelic case is reformulated as a sequence of “biallelic” tests. However, in this approach it is not obvious how to assess the general evidence of the original hypothesis; nor is it clear how to establish the significance level for its acceptance/rejection. In this work, we introduce a straightforward method for the multiallelic HWE test, which overcomes the aforementioned issues of sequential methods. The core theory for the proposed method is given by the Full Bayesian Significance Test (FBST), an intuitive Bayesian approach which does not assign positive probabilities to zero measure sets when testing sharp hypotheses. We compare FBST performance to Chi-square, Likelihood-ratio and Markov chain tests, in three numerical experiments. The results suggest that FBST is a robust and high performance method for the HWE test, even in the presence of several alleles and small sample sizes.

## Introduction

The Hardy-Weinberg law is one of most important principles in population genetics, and establishes a direct relationship between allele and genotypic proportions in a population. This law states that in a large population of panmictic dioecious organisms with non-overlapping generations, the allelic and genotypic frequencies at a locus will stay unchanged, provided that migration, mutation, and natural selection do not affect that locus. When these conditions hold, it is said that the locus is under Hardy-Weinberg Equilibrium (HWE).

This principle was discussed independently by Yule, Pearson and Castle (between 1902 and 1904), for some particular allele frequencies (see references in Crow and Kimura, 1972). In 1908, Godfrey Hardy presented the general principle for two alleles (Hardy, 1908). This principle was called Hardy's law for 35 years, until Stern (1943) called attention to an article of Weinberg (1908) showing the same principle at the same time and demonstrating its validity for multiple alleles (Crow, 1999).

Since its postulation, several results in population genetics and much forensic inference based upon DNA evidence have been based on the assumption that HWE is valid for the genetic loci of interest. Some statistical tests to detect and measure deviation from HWE have been devised, and their limitations have become more obvious when testing for deviation within multiallelic DNA loci. The most common approach consists of goodness-of-fits tests, like Chi-square and Likelihood-ratio, which are heavily based on asymptotic results, and can sometimes lead to false rejection or acceptance of HWE when the sample sizes are small and/or some genotype sample frequencies are very small (Emigh, 1980). Another approach involves exact tests, but is restricted to small dimensions and allele numbers.

A Bayesian sequential method for multiallelic HWE test was proposed by Pereira *et al.* (2006), who suggested reformulating the multiallelic case as a sequence of “biallelic” tests. In that work, the central component is the Full Bayesian Significance Test (FBST), an intuitive Bayesian approach which does not assign positive probabilities to zero measure sets when testing sharp hypotheses (Pereira and Stern, 1999). Although the sequential method avoids the quadratic increase of parameter space dimension with respect to the number of alleles, it is not obvious how to assess the general evidence of the original hypothesis; nor is it clear how to establish the significance level for its acceptance or rejection (see DeGroot, 1970).

In this work, we propose a method for the multiallelic HWE test, based on the FBST. FBST has many theoretic and practical advantages over other approaches, and it has shown to be robust in several high-dimensional problems (Lauretto *et al.*, 2008).

## Background

In this section we introduce some notations, and the Hardy-Weinberg Equilibrium (HWE) formula. Let us consider *k* alleles *A*_1_, *A*_2_, ..., *A*_*k*_ in a *locus*. The main interest is to assess the population relative frequencies of the genotypes *A*_*i*_*A*_*j*_ (*i, j* = 1, 2, ..., *k*) which we denote by *p*_*ij*_. As usual in the literature (see Hardy, 1908), we assume that the allele frequencies do not depend on sex and thus are symmetric, that is, *A*_*i*_*A*_*j*_ is equivalent to *A*_*j*_*A*_*i*_ and *p*_*ij*_ = *p*_*ji*_. Therefore, the parameter of interest is the (lower triangular) matrix of genotype proportions:

θ = (θ_*ij*_), with θ_*ii*_ = *p*_*ii*_, θ_*ij*_ = 2*p*_*ij*_ for 1 ≤ *j* ≤ *i* ≤ *k*.

We denote by *p*_1_, *p*_2_, ..., *p*_*k*_ the (unknown) population frequencies of alleles *A*_1_, *A*_2_, ..., *A*_*k*_, with *p*_i_ ≥ 0 and 
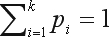
. When the locus is under HWE, the genotype proportions are as follows:


(1)
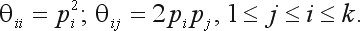


In order to test the HWE in a locus, one considers a sample of *n* individuals drawn randomly from the population. Such a sample can be presented as the array *x*, whose elements 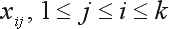, are counts of genotypes *A*_*i*_*A*_*j*_. The sample size *n* is 
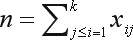
, and the sample frequency of allele *A*_*i*_ is 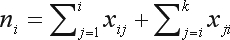. Note that 
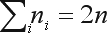
, since all loci have two alleles and this sum is the number of alleles in the whole sample. The sample proportion of allele *A*_*i*_, 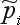, is given by


(2)
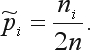


Assuming that each individual genotype does not depend on remaining individuals in the same generation, we can consider that the genotype frequencies *x*_*ij*_ follow a multinomial distribution with unknown parameter θ,


(3)
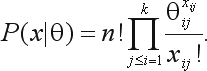


## Testing Procedures

In this section we present three tests used in our comparative study. These and other approaches are described by Emigh (1980), Guo and Thompson (1992), Hernández and Weir (1989) and Montoya-Delgado *et al.* (2001).

###  Chi-square goodness-of-fit test

This test involves calculating the sample chi square value,


(4)
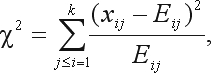


with



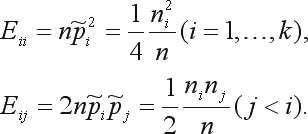


Under HWE, this quantity has a chi-square distribution with *k*(*k* – 1)/2 degrees of freedom.

The Chi-square goodness-of-fit test with continuity correction involves calculating the previous statistics, with the subtraction in each term of a correction constant *c*:


(5)
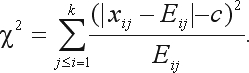


Usually *c* = 0.5 is the value chosen.

###  Likelihood-ratio tests

The likelihood function, given a sample, follows directly from the multinomial distribution presented in Eq. (3). A Likelihood-ratio test is constructed by comparing the likelihood maximized under the hypothesis, *L*_0_, with the maximum likelihood, *L*_1_, not constrained by the hypothesis. For HWE we have


(6)
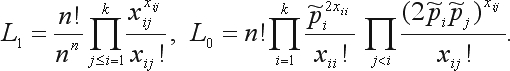


with the sample allelic frequencies, 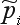, given by Eq. (2).

The test statistic


(7)
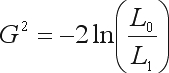


is asymptotically distributed as a chi-square distribution with *k*(*k* – 1)/2 degrees of freedom.

###  Markov Chain Monte Carlo (MCMC) method

Proposed by Guo and Thompson (1992), the method consists of an adaptation of the Metropolis algorithm, with the construction of a Markov chain with equilibrium distribution matching the genotype probabilities under HWE of samples that have the same allelic counts as the observed data.

Under HWE and conditional on sample allele counts, *n*_1_, *n*_2_, ..., *n*_*k*_, the probability of obtaining the sample *x* is (see Levene, 1949):


(8)
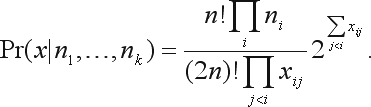


Given the data *x*, the test evaluates


(9)
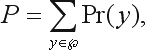


where 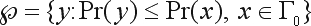 and


(10)
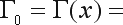
 {*y*: *y* has the same allele counts as does *x*}.

The MCMC algorithm is performed in order to estimate the probability *P* in E. (9). Rejection or acceptance of the null hypothesis depends on whether *P* is smaller than a pre-specified significance level α.

## Methods

###  The Full Bayesian Significance Test (FBST)

The Full Bayesian Significance Test (FBST) was proposed by Pereira and Stern (1999) as a coherent and intuitive Bayesian test. It assumes that the hypothesis, *H*, is defined as a subset defined by inequality and equality constraints:


(11)
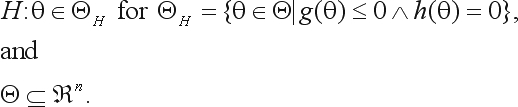


For simplicity, we often use *H* for Θ_*H*_. FBST is particularly focused on precise hypotheses: *i.e*., dim(Θ_*H*_) < dim(Θ). In this work, 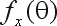 denotes the posterior probability density function, given the observation *x*. Bold **0** and **1** denote vectors of appropriate dimensions.

For the HWE test, the parametric space consists of all arrays of genotype proportions


(12)
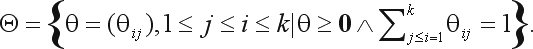


The space on hypothesis is


(13)
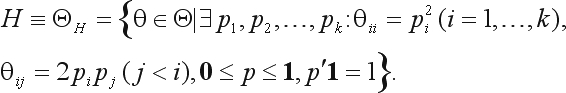


As previously stated, we consider that the genotype frequencies *x* follow a multinomial distribution, given by Eq. (3). Taking as a priori the Dirichlet distribution with parameters (1,1...1), *i.e.*, a uniform distribution, then the a posteriori is a Dirichlet distribution with parameters (*x*_11_ + 1, *x*_21_ + 1, *x*_22_ + 1, ..., *x*_*kk*_ + 1) which is proportional to the likelihood function (DeGroot, 1970):


(14)
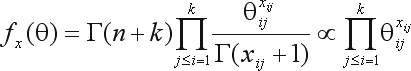


The computation of the evidence measure used on the FBST is performed in two steps:

1. *The optimization step* consists of finding the maximum (supremum) of the posterior under the null hypothesis, 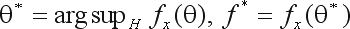.

2. *The integration step* consists of integrating the posterior density over the Tangential Set, $\bar{T}$, where the posterior is higher than anywhere in the hypothesis, *i.e.*,


(15)
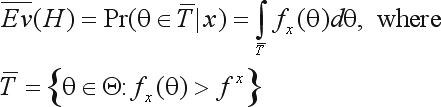


**Figure 1:**
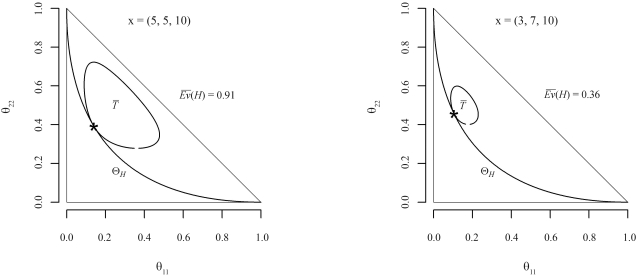
Parameter space representation for two biallelic examples, *x* = (5,5,10) and *x* = (3,7,10), and the respective evidence against the HWE hypothesis.

$\bar{E}v(H)$ is the evidence against *H*, and $\bar{E}v(H)$= 1 – $\bar{E}v(H)$is the evidence supporting (or in favor of) *H*. For a better understanding of this evidence measure, Figure 1 illustrates two examples in the biallelic case, showing the null and tangential sets (Θ_*H*_ and $\bar{T}$).  Since θ_21_ = 1 – θ_11_ – θ_22_, the parametric space is fully defined by homozygote proportions, θ_11_ and θ_22_. The parameter space corresponds to the area inside the triangle. Sample genotype counts for *A*_11_, *A*_21_, *A*_22_ and $\bar{E}v(H)$are also shown in each graph. Marker ‘*' represents the point θ^*^ of maximum a posteriori density in the constrained space Θ_*H*_, and the level curve tangent to θ^*^ corresponds to $\bar{T}$frontier. Intuitively, if the hypothesis set is in a region of “low” posterior density (as in the example 1), then $\bar{T}$is “heavy” and therefore $\bar{E}v(H)$is “large” (≅ 0.91), meaning “strong” evidence against *H*. On the other hand, as illustrated by the example 2, if hypothesis set is in a region of “high” posterior density, then $\bar{T}$is a “small” set, and hence *Ev*(*H*) is “small” (≅ 0.36), meaning “weak” evidence against *H*.

For HWE test, the point 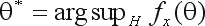 is given as follows.

Rewriting the posterior pdf under HWE, we have:


(16)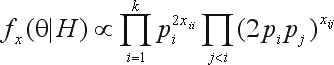.

Taking its logarithm,


(17)
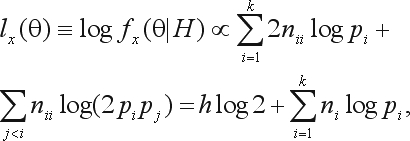


where 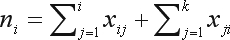, and *h* is the sum of sample heterozygote frequencies, 
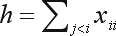
.

By the constraint 
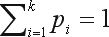
, we have:


(18)
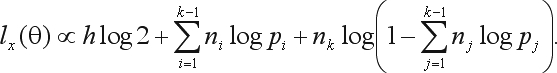


The gradients of *l*_*x*_(θ) are given by:


(19)
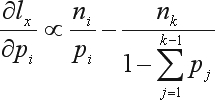


Hence, the optimal point under HWE is given by the vector 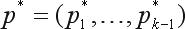 which satisfies:


(20)
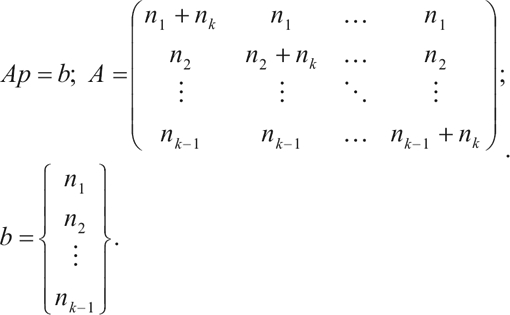


Summing over all constraints and after some algebra, we obtain the following solution:



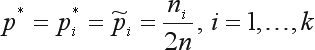
.

The computation of θ^*^ from p^*^ follows from Eq. (1).

The integration step may be performed by generating a set of *M* points 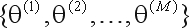 with a Dirichlet distribution with parameter (*x*_11_ + 1, *x*_21_ + 1, *x*_22_ + 1, ..., *x*_*kk*_ + 1) and computing the percentage of points with posterior density greater than f^*^:


(21)
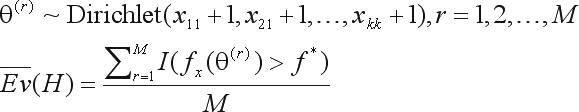


where *I*(*stat*) = 1 if *stat* is true and 0 otherwise. A more precise and efficient Monte Carlo method for the integration step is presented by Lauretto *et al.* (2003).

As with any significance test, this procedure requires the choice of a threshold level, τ, for acceptance/rejection of the hypothesis at a significance level α. Several alternative methods have been developed for establishing this threshold:

- An empirical power analysis, developed by Stern and Zacks (2002) and Lauretto *et al.* (2003), provides critical levels that are consistent and also effective for small samples.
- A threshold based on reference sensitivity analysis and paraconsistent logic is given by Stern (2004).
- Pereira *et al.* (2008) relates the e-value threshold to standard p-value thresholds.
- Madruga *et al.* (2001) proves the existence of a loss function that renders the FBST a true Bayesian decision-theoretic procedure.
- An asymptotically consistent threshold for a given confidence level was given by Stern (2007), and Borges and Stern (2007), which we adopt in this work.

Let us consider the cumulative distribution of the evidence value against the hypothesis, 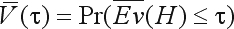, given θ^0^, the true value of the parameter. Under appropriate regularity conditions, for increasing sample size, we can state the following:

If *H* is false, 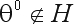, then $\bar{E}v(H)$converges (in probability) to one, that is, 
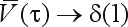
.

If *H* is true, 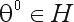, then $\bar{V}(\tau )$, the confidence level, is approximated by the function *Q*(*t*, *h*, τ) = 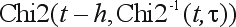, where *t* = dim(Θ), *h* = dim(*H*) and Chi2(*df*, *x*) denotes the cumulative chi-square distribution with *df* degrees of freedom.

Hence, to reject *H* with a significance level α, we can set 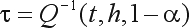, *i.e.*, set τ such that 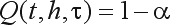.

## Results and Discussion

**Figure 2:**
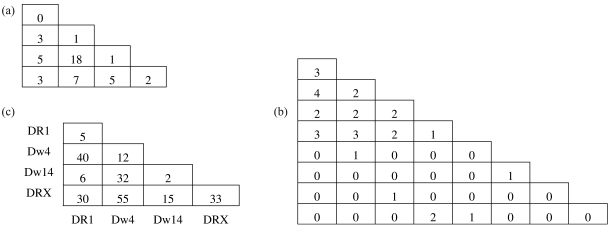
Datasets for numerical experiments, given by Louis and Dempster (1987) (a), Guo and Thompson (1992) (b) and Wordsworth *et al.* (1992) (c).

The numerical experiments used in the performance analysis are based on three typical datasets. Two examples consist of simulated data used in the literature as benchmarks for comparing the performance of competing methods, while the third example is from a real dataset. These examples are presented in Figure 2, as lower triangular matrices containing genotype frequencies. The first example is taken from Louis and Dempster (1987) and consists of a sample of size 45 of genotype frequencies distributed in four alleles. The second example is given by Guo and Thompson (1992) and consists of a sample of size 30 of simulated genotype frequencies simulated under HWE with underlying allele frequencies (0.2, 0.2, 0.2, 0.2, 0.05, 0.05, 0.05, 0.05). The third example is from a rheumatoid arthritis (RA) study performed by Wordsworth *et al.* (1992), where two hundred and thirty RA patients were genotyped for the HLA-DR locus. The DR4 allele was subdivided into Dw4, Dw14 and other subtypes. DRX represents all non-DR1, non-Dw4, non-Dw14 alleles. This example is used by Chen and Thomson (1999) as a benchmark.

Our main interest is to compare the error convergence of FBST and other methods presented in this work (MCMC, Chi-square and Likelihood-ratio) for increasing sample sizes. For each sample size 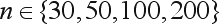, we simulated two collections of 100 samples. The first collection consists of samples drawn under HWE, *i.e.*, each sample is drawn with a multinomial distribution with parameters (*n*, θ^*^), with 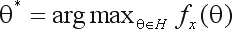. The second collection consists of samples drawn with a multinomial distribution with parameters (*n*, θ^(^^*h*^^)^), where θ^(^^*h*^^)^ is drawn under the posterior distribution. That is, each sampling iteration is performed in two steps:

a) draw 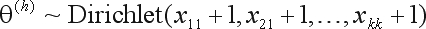, where *x*_*ij*_ (1 ≤ *j* ≤ *i* ≤ *k*) are the frequencies in the original dataset; and

b) draw 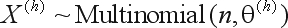.

The Type I error (rejection rate of a true hypothesis) is estimated by the proportion of samples in collection 1 such that HWE is rejected, and the Type II error (acceptance rate of a false hypothesis) is estimated by the proportion of samples in collection 2 such that HWE is accepted. The performance criterion used in this work is the average error, *i.e.*, the average of Types I and II error rates. Two standard significance levels, α ∈ {0.01, 0.05}, were used to calibrate the asymptotic acceptance/rejection threshold of each method.

A variability measure for the errors was obtained by performing 10 batches of simulations and computing the mean and standard deviation of average errors across the batches.

**Figure 3:**
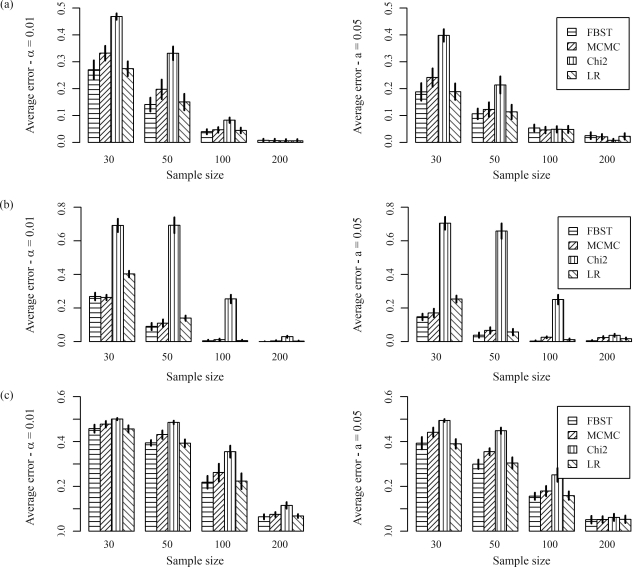
Average error rates for FBST, MCMC, Chi-square and Likelihood-ratio for simulated data based on examples from Louis and Dempster (1987) (a), Guo and Thompson (1992) (b) and Wordsworth *et al.* (1992) (c).

Figure 3 presents the average errors for FBST, MCMC, Chi-square (with continuity correction) and Likelihood ratio for simulated data based on examples 1, 2 and 3. The bar height represents the mean of average errors, and the vertical line on the top of each bar is the error bar, representing the mean ± one standard deviation of average errors.

For simulated data based on examples 1 and 3, the best competitors are FBST and Likelihood-ratio test, while for simulated data based on example 2, the best competitors are FBST and MCMC. In every case, we notice that FBST is always the best competitor (especially for small sample sizes, *n* ≤ 100) or is very close to it.

These numerical results suggest that FBST is more stable than the competitors discussed in this paper, in the sense that it has good comparative performance for different datasets and allele numbers.

## Final Remarks

We have introduced a simple and straightforward procedure for testing deviance from Hardy-Weinberg Equilibrium (HWE) in the presence of several alleles. This procedure was implemented in C language, and integrated into a system for parentage testing developed with FAPESP support, where it is applied in the selection of loci to be used for parentage testing. Further details of this project can be found at http://watson.fapesp.br/PIPEM/Pipe13/genet1. htm. Currently, the routine is available by request to the corresponding author.
